# Current status of room temperature magnetic compensation in impurity-doped Mn_4_N epitaxial thin films

**DOI:** 10.1080/14686996.2026.2688056

**Published:** 2026-06-19

**Authors:** Tomohiro Yasuda, Takashi Suemasu

**Affiliations:** a Degree Programs in Pure and Applied Sciences, Graduate School of Science and Technology, University of Tsukuba, Tsukuba, Ibaraki, Japan; b Department of Applied Physics, Institute of Pure and Applied Sciences, University of Tsukuba, Tsukuba, Ibaraki, Japan

**Keywords:** Ferrimagnet, Mn_4_N, magnetic compensation, XAS, XMCD, AHE

## Abstract

Mn_4_N, a ferrimagnet that does not contain rare-earth elements, possesses attractive properties for spintronics applications. In particular, Mn_4_N epitaxial films exhibit a small spontaneous magnetization of about 100 kA m^−1^, a large spin polarization (*p* = 0.8), and large perpendicular magnetic anisotropy with a magnetic anisotropy constant of approximately 10^5^ J m^−3^. More importantly, achieving magnetic compensation at room temperature is possible through impurity doping. This property is particularly significant for spin–torque-based spintronics applications. In the vicinity of the magnetization and/or angular momentum compensation points, magnetization dynamics can be substantially accelerated, enabling high-speed switching and domain wall motion. This article surveys the magnetic properties of impurity-doped Mn_4_N epitaxial films grown on SrTiO_3_(001) substrates, highlighting recent results obtained with Cu-, Ag-, Au-, and Pd-doped Mn_4_N films in comparison with Ni- and Cr-doped Mn_4_N films. Furthermore, with a view to device applications of Mn_4_N, we present the formation of ultrathin (~4 nm) Pt epitaxial films with a <100> orientation on MgO (001), which is essential for injecting spins into Mn_4_N-based overlayers by utilizing the spin Hall effect of heavy metals.

## IMPACT STATEMENT

This paper summarizes the latest results on magnetization compensation in impurity-doped Mn_4_N epitaxial films and the formation of <1 0 0>-oriented ultrathin Pt epitaxial films on MgO (0 0 1) surfaces for integration with MTJs.

## GRAPHICAL ABSTRACT

**Figure:**
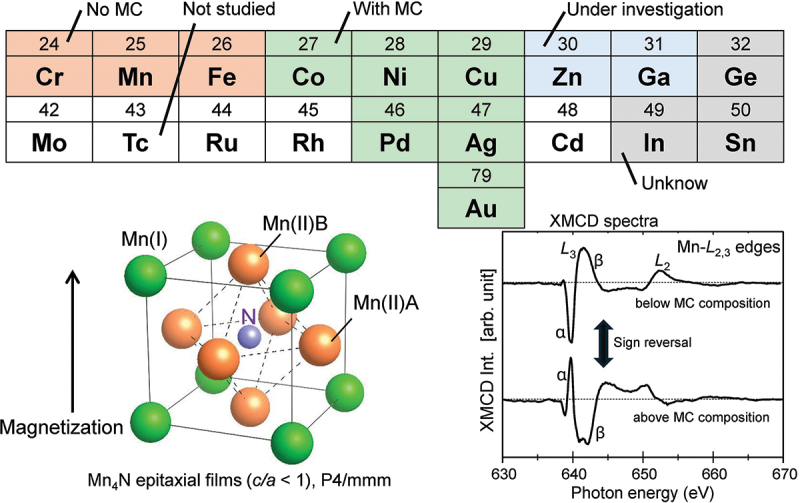

## 1. Introduction

**Figure 1.**
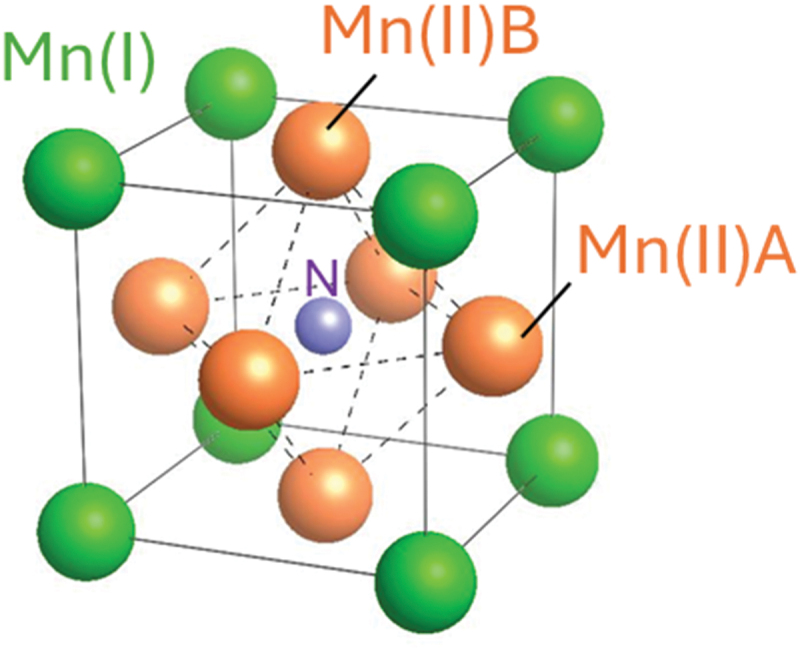
Crystal structure of anti-perovskite Mn_4_N.

In spintronics, one of the key areas of research is how efficiently spin precession and reversal can be induced using spin-transfer torque (STT) and spin-orbit torque (SOT) [1–3]. Under such circumstances, ferrimagnets (FIMs) have garnered increasing attention [4–22]. The main characteristic of ferrimagnets is that the magnetic moments of different sublattices arrange so as to partially compensate each other. Consequently, unlike antiferromagnets, ferrimagnets retain an imbalance between the magnetic moments of different sublattices, leading to a finite spontaneous magnetization that is much smaller than that of ferromagnets. This property offers a significant advantage in the development of application technologies based on STT and SOT, where the angular momentum carried by the incident spin currents acts on the magnetization, inducing precession or reversal of the magnetization. In the case of STT, the spin-polarized current is generated within the magnetic layer by exchange interactions, while in the case of SOT, a pure spin current can often be generated by the spin Hall effect (SHE) within a heavy-metal layer in contact with the magnetic layer. It is also generated by the Rashba-Edelstein effect at interfaces that break inversion symmetry or in topological insulators. In either case, the torque arises from the conservation of angular momentum. When a ferrimagnet is close to an angular momentum compensation (AC) and/or a magnetic compensation (MC) point, the angular momentum carried by spin currents readily affects the reduced magnetization, resulting in a high-speed magnetization dynamics [12,14,19]. Therefore, ferrimagnets are attractive materials for the development of devices based on STT and SOT, such as domain-wall memory, three-terminal magnetic random-access memory, and devices utilizing skyrmion motion [23–25]. The most commonly used approach to achieve MC and AC in spintronics is based on alloys combining rare earth elements and transition metals, such as Gd_0.44_Co_0.56_, Tb_0.21_Co_0.79_, and Gd_3_Fe_5_O_12_ [5,6,10,12,13,20]. When the magnetizations of the two species are antiparallel, there exists a certain concentration at which compensation occurs at room temperature (RT). One of such materials is rare-earth-free Mn_4_N [26–31], and thereby we have focused on this material [32,33]. As shown in Figure 1, Mn_4_N has an anti-perovskite structure. It consists of two sublattices: one containing Mn(I) at the corner sites and the other containing Mn(II) at the face-centered sites. Note that there are two sites, A and B, for Mn(II) in Mn_4_N epaxial films (crystallographic space group; *P*4/*mmm*) since they are subjected to tensile stress in the in-plane direction (*c*/*a* < 1) [34], differently from Mn_4_N bulk crystals (crystallographic space group; Pm$\overset{ˉ}{3}$m). Extensive research has been conducted on Mn_4_N-based mixed crystals, particularly on Mn_3_AN, a non-collinear antiferromagnet, and a wide variety of studies are summarized in Ref [35]. Detailed analyses of Mn_4_N bulk crystals revealed that the noncollinear frustrated moments of Mn(II) sites of the (111) kagome planes tilt about 20° out-of-plane and are easily influenced by the substitutions on Mn(I) sites [28]. This study also discusses the mechanism by which impurities induce MC. Since the observation of perpendicular magnetic anisotropy (PMA) in Mn_4_N films [36–38], interest in Mn_4_N-based epitaxial thin films has been growing together with the magnetic structures in Mn_4_N films, which is different from the triangular ferrimagnetism of the bulk [39,40]. Mn_4_N epitaxial thin films have attracted attention in the field of spintronics due to the following characteristics.

- A relatively large PMA and a small spontaneous magnetization (*M*_S_ ~ 100 kA m^−1^) have been reported in Mn_4_N films deposited on various substrates, including glass, MgO (001), Si (001), LaAlO_3_[LAO] (001), (LaAlO_3_)_0.3_(Sr_2_TaAlO_6_)_0.7_(001), and SrTiO_3_[STO] (001) [34,36,37,41–44]. The uniaxial magnetic anisotropy constant ($K_{u}$) was reported to be of the order of 10^5^ J m^−3^ for Mn_4_N thin films [32].
- The effective spin polarization derived from the domain wall (DW) velocity measurements in Mn_4_N strips is relatively high (*p* = 0.8) [45]. This enhances magnetic transport effects utilized in spintronics, such as the anomalous Hall effect (AHE) and the giant magnetoresistance, suggesting that Mn_4_N could be used as a magnetic soft layer in magnetic tunnel junction stacks.
- The magnetization reversal is caused by nucleation and the motion of Bloch DWs. The combination of a small *M*_S_ and a relatively large *P* results in higher DW velocities due to the STT [19,45]. The current-induced DW velocity due to the adiabatic torque is expressed by
  (1) $$v\approx \frac{1}{1+\alpha^{2}}\frac{g\mu_{B}}{2eM_{S}}\mathrm{PJ}\propto \frac{P}{M_{S}}.$$

Here, *v* is the DW velocity, *α* is the damping constant, *g* is the Landé factor, *e* is the electron charge, and *J* is the current density. The interest of the small magnetization is both for STTs through magnetic tunnel junction and for SOTs.

iv) The combination of a high PMA and a moderate exchange stiffness results in a relatively sharp Bloch wall. A sharp DW can enhance the spin transfer effect through non-adiabatic contributions [46] and may contribute to the rapid motion of the DW at smaller currents [47]. The width (*Δ*) of the DW in a nanowire is given by Eq. (2) [48],

(2) $$\Delta =\sqrt{A_{\mathrm{stiff}}/K_{u}}.$$

Here, $A_{\mathrm{stiff}}$ is the exchange stiffness. As $K_{u}$ increases, *Δ* decreases, and the threshold current density (*j*_th_) required to move the DWs decreases [47,49]. In this regard, materials with PMA are advantageous for current-induced domain wall motion (CIDWM) devices. Furthermore, to scale down DW-based logic devices [50], three terminal memories [51], or racetrack memories [52] to the ultimate technology node, the DW width must be sufficiently reduced.

This paper outlines our research group’s recent findings on impurity-doped Mn_4_N epitaxial films. Mainly focusing on experimental results from X-ray absorption spectroscopy (XAS), X-ray magnetic circular dichroism (XMCD), and AHE loop measurements, we investigate the presence or absence of MC at RT in Mn_4_N epitaxial films doped with various impurities (Ni, Cu, Ag, Au, and Pd). Finally, with a view to device applications of Mn_4_N on MgO (001) surfaces, we present the formation of approximately 4 nm thick <1 0 0>-oriented Pt epitaxial films on MgO (001) substrates. This is essential for injecting spin into the Mn_4_N-based film by utilizing the SHE of heavy metals.

## 2. Methods

### 2.1. Thin film growth

Mn_4_N films have been grown on various substrates by conventional thin-film growth techniques like molecular beam epitaxy (MBE), sputtering, and others [35]. Instead of the MgO (001) substrates commonly used in spintronics, we employed STO (0 0 1) substrates, which exhibit minimal lattice mismatch with Mn_4_N. This was done to grow high-quality Mn_4_N epitaxial films and to clearly demonstrate the effects of impurity doping [32]. The lattice constant of MgO, STO, and Mn_4_N is 0.421 nm, 0.391 nm, and 0.387 nm, respectively. Before deposition, the STO substrates were etched for 40 s in buffered hydrofluoric acid. This was done to create a step-and-terrace surface structure, thereby ensuring a surface that was atomically smooth and terminated with Ti [53]. Approximately 10–25-nm-thick impurity doped Mn_4_N layers were grown on STO (0 0 1) substrates at a substrate temperature of around 450°C by MBE. The optimum substrate temperature during thin-film deposition may vary depending on the type of impurity. Metal elements including Mn were supplied by Knudsen cells, and nitrogen was supplied using a radio-frequency (RF) plasma source. To prevent oxidation, the sample surfaces were covered with sputter-deposited 2–3-nm thick capping layers like SiO_2_, Pt, and others.

### 2.2. Characterizations of film properties

The crystalline quality of the grown films was characterized by 20-kV reflection high-energy electron diffraction (RHEED) and X-ray diffraction (XRD; Rigaku SmartLab) with a Cu *K*α radiation source. The composition ratio of Mn to doped impurity element was measured by energy dispersive X-ray spectroscopy (EDX, HITACHI SU7000) or just by their deposition rates. AHE loops were acquired with a physical properties measurement system (PPMS, Quantum Design, Inc.) using the Van der Pauw method. The transverse voltage (*V*_y_) is expressed as Eq. (3) [54]:

(3) $$V_{y}=\left(R_{H}\frac{B_{z}}{t}+\frac{\rho_{\mathrm{AHE}}}{t}\right)I_{x}=\frac{\rho_{\mathrm{yx}}}{t}I_{x}.$$

Here, $R_{H}$, $B_{z}$, *t*, $\rho_{\mathrm{AHE}}$, *I*_*x*_, and $\rho_{\mathrm{yx}}$ are the normal Hall coefficient, the magnetic flux density perpendicular to the sample surface, the film thickness, the anomalous Hall resistivity, the longitudinal current, and the Hall resistivity, respectively.

X-ray absorption spectroscopy (XAS) and X-ray magnetic circular dichroism (XMCD) measurements were conducted at beamline BL-16A of KEK-PF, Japan. During the measurement, circularly polarized soft X-rays were incident. XMCD spectra were acquired in a single scan, while switching between left and right polarizations at a frequency of 10 Hz using two undulators. The spectra at the Mn-*L*_2,3_ absorption edges were measured by the total electron yield (TEY) method. All measurements were performed at RT, either in the saturated magnetization state at 3‒5 T or in the remanent magnetization state at 0 T. To minimize systematic errors arising from the measurement system, the sign of the XMCD spectrum obtained at *μ*_0_*H* = −5 T was inverted, and the sum of this spectrum and the XMCD spectrum obtained at *μ*_0_*H* = +5 T was adopted as the spectrum obtained at 5 T. Similarly, the XMCD spectrum at 0 T consists of two spectra: one obtained by increasing the external magnetic field from *μ*_0_*H* = −5 T to −0 T, and the other obtained by decreasing the external magnetic field from *μ*_0_*H* = +5 T to + 0 T. The X-ray absorption near edge structure (XANES) measurements were performed in the fluorescence yield mode (TFY) at BL08W of the NanoTerasu facility, targeting the Pd and Ag-*L*_3_ edges. All measurements were performed at RT. The experimental data were processed to eliminate the backgrounds and normalize the spectra using Athena, an X-ray absorption spectroscopy (XAS) processing system [55]. The XANES simulations were conducted using the finite-difference method for near-edge structure (FDMNES) codes [56].

## 3. Results and discussion

### 3.1. XAS and XMCD spectra of Mn_4_N epitaxial films

**Figure 2.**
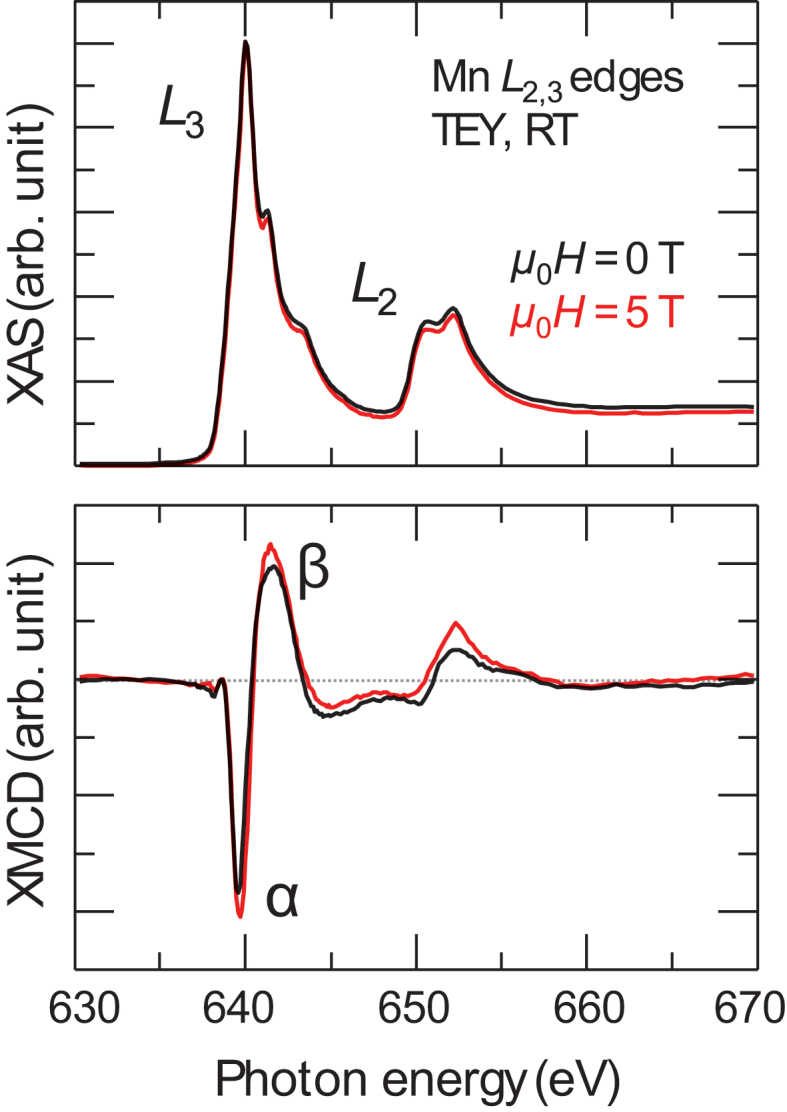
XAS and XMCD spectra of Mn_4_N epitaxial films measured at the Mn-*L*_2,3_ edges at *μ*_0_*H* = 0 T and 5T. The spectrum at 0 T is obtained by first applying an external magnetic field up to 5 T, then lowering the field to 0 T, and finally performing the measurement.

**Figure 3.**
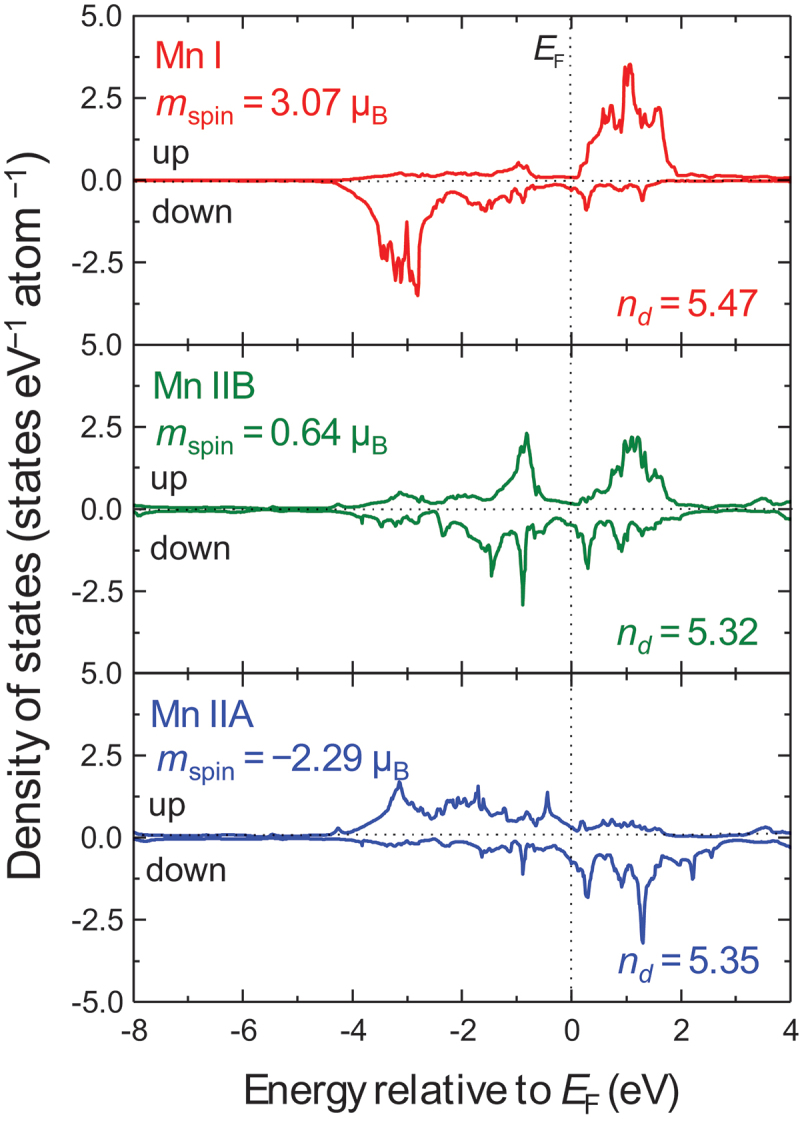
Total density of states of Mn (I), Mn(II)A, and Mn(II)B in Figure 1 [38].

Figure 2 shows typical examples of XAS and XMCD spectra measured for an Mn_4_N epitaxial film on STO (001) at the Mn-*L*_2,3_ edges under external magnetic fields of *μ*_0_*H* = 0 T and 5 T applied normal to the sample plane. About 5 T was sufficiently high to saturate the magnetization. The spectra at *μ*_0_*H* = 0 T and 5 T are nearly identical. The XMCD spectrum of the Mn_4_N film shows a sharp peak (peak α) and a broad peak (peak β) with the opposite sign. Figure 3 shows the theoretical density of states of Mn_4_N by the all-electron full-potential linearized augmented-plane-wave method [57]. Narrow and wide bandwidths are obtained for Mn(I) and Mn(II) atoms, respectively. This is because the Mn(I) atoms at the corner sites are farther from the nitrogen atoms in the body-centered lattice, making it more difficult for them to form hybridized orbitals compared to the Mn(II) atoms at the face-centered sites. As a result, the electrons of the Mn(I) atoms are relatively localized and are thought to contribute primarily to the sharp peak α. On the other hand, the broad peak β is primarily caused by the itinerant electrons of the Mn(II) atoms at the face-centered sites, due to hybridized orbitals formed with nitrogen [57]. Therefore, the fact that the signs of peak α and peak β are opposite reflects that the magnetic moments of the corner-site Mn(I) sublattice and the face-centered Mn(II) sublattice are oriented in opposite directions, and the magnetic moment of the Mn(I) sublattice is parallel to the magnetization. Using the Bohr magneton (*μ*_B_), the calculated spin magnetic moment for the Mn(I) is 3.07 *μ*_B_/atom, and those for the Mn(II) at inequivalent A and B sites are −2.29 *μ*_B_/atom and 0.64 *μ*_B_/atom, respectively. This means that the Mn(I) moment is aligned antiparallel to the magnetic filed, which is opposite to what was observed in the XMCD spectra (Figure 2). This suggests that even for the metallic Mn_4_N, more advanced modeling is required that takes into account the core hole-3d and 3d–3d interactions. Another possible factor is that defects present in the thin films formed by the experiments may have an influence. This could be the reason why the experimentally obtained XMCD spectra differ from the predictions derived from first-principles calculations. However, as will be discussed later, when performing XAS and XMCD measurements on Mn_4_N thin films doped with impurity elements, we always use Mn_4_N epitaxial thin films as the reference sample. Consequently, the results shown in Figure 2 have been consistently obtained; we have never obtained results in which the signs of peaks α and β are reversed, as predicted by theoretical calculations, nor have we found any reports of such results. Anyway, now that we understand the characteristics of XAS and XMCD spectra of MBE-grown Mn_4_N epitaxial films, we will next discuss the results of impurity doped Mn_4_N epitaxial films.

### 3.2. Magnetic compensation in Ni-doped Mn_4_N epitaxial films

**Figure 4.**
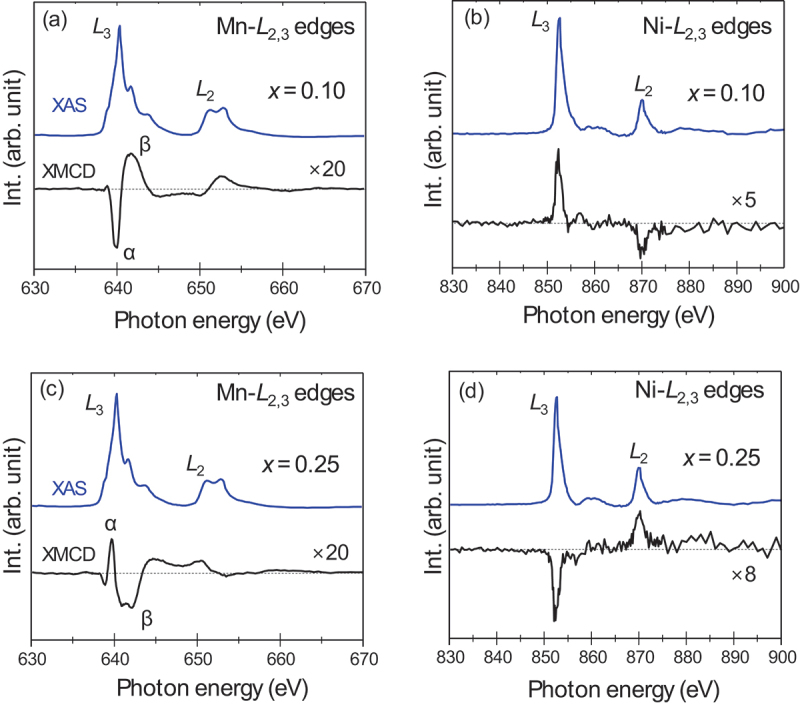
XAS (blue) and XMCD (black) spectra measured at (a)(c) Mn-*L*_2,3_ and (b)(d) Ni- *L*_2,3_ edges for (a)(b) Mn_3.9_Ni_0.1_N films and (c)(d) Mn_3.75_Ni_0.25_N films [26]. Reproduced by permission from [26], copyright [2020, AIP Publishing].

Figure 4 shows the XAS and XMCD spectra of the Mn- and Ni-*L*_2,3_ edges of Ni-doped Mn_4_N epitaxial films (Mn_4−*x*_Ni_*x*_N) formed on STO (001) under conditions where an external magnetic field of *μ*_0_*H* = 3 T was applied perpendicularly normal to the sample plane [26]. The Ni composition *x* was determined from the deposition rates of Mn and Ni. In the XAS spectrum of Ni, as there is no shoulder peak appearing 2 eV higher than the main peak, which would be expected if Ni occupies an Mn(II) site. Therefore, it is thought that Ni substituted for an Mn(I) site, *i.e*., it is Ni(I). Since there was no significant difference in the XAS spectra of the Mn- and Ni-*L*_2,3_ edges regardless of the value of *x*, we focus here on the XMCD spectra. The XMCD spectrum of the Mn-*L*_2,3_ edges for the *x* = 0.10 sample, shown in Figure 4(a), is similar to that for the Mn_4_N film in Figure 2, meaning that the magnetic moment of the Mn(I) sublattice is aligned parallel to the magnetization at *x* = 0.10. In contrast, the sign of the XMCD signal of the Ni-*L*_3_ edge in Figure 4(b) is opposite to that of peak α in Figure 4(a). This result indicates that the magnetic moment of Ni(I) is oriented antiparallel to the magnetization. The element-specific magnetic moment can be calculated using the sum-rule analysis [58,59]. Table 1 summarizes the spin (*m*_spin_), orbital (*m*_orb_), and total (*m*_tot_ = *m*_spin_ + *m*_orb_) magnetic moments per Mn and Ni atom for samples at *x* = 0.10 and 0.25. For the number of holes in Mn and Ni atoms, 5.2 was adopted for Mn [60] and 1.4 for Ni [61], regardless of whether they were at I or II sites. For the *x* = 0.1 sample, as suggested by the sign reversal in Figure 4(b), the magnetic moment of Ni(I) is negative; therefore, the addition of Ni causes a decrease in magnetization. When the Ni composition was increased further to *x* = 0.25, the sign of the XMCD signals of Mn and Ni reversed compared to that at *x* = 0.10, and the magnetic moment of the Mn(II) sublattice is parallel to the magnetization. Furthermore, the magnitude of *m*_tot_ for Ni remained almost unchanged between *x* = 0.1 and 0.25 in Table 1, suggesting that the preferred occupancy sites of the Ni atoms did not change. These results are consistent with the aforementioned analysis based on the XMCD spectrum. We therefore conclude that the MC composition (*x*_MC_) in Mn_4−*x*_Ni_*x*_N lies between 0.10 and 0.25. The Ni composition *x* = 0.10 is considered very close to *x*_MC_. This is because the *m*_tot_ per unit cell was as small as approximately 0.009 *μ*_B_ at *x* = 0.10 and increased to approximately 0.39 *μ*_B_ at *x* = 0.25. These values correspond to approximately 1.5 kA m^−1^ and 63 kA m^−1^, respectively. Furthermore, the coercive force of the MOKE hysteresis loop was approximately four times that at *x* = 0.25. Similar sign reversal of the XMCD spectra were also observed at the Mn-*L*_2,3_ and Co-*L*_2,3_ edges in the Mn_4-x_Co_x_N epitaxial films [27,57], and the results can be explained by the view that Co first substitutes for Mn(I) sites and then for Mn(II) sites as the Co concentrations increases [27].

**Table 1.** Spin (*m*_spin_), orbital (*m*_orb_), and total (*m*_tot_) magnetic moments of Mn and Ni atoms in Mn_4−*x*_Ni_*x*_N deduced by sum-rule analysis.

Compounds	Atom	Magnetic moment [*μ*_B_ per atom]		
		*m*_spin_	*m*_orb_	*m*_tot_
Mn_3.9_Ni_0.1_N	Mn Ni	0.024 ± 0.011 −0.20 ± 0.03	−0.016 ± 0.002 −0.024 ± 0.004	0.008 ± 0.014 −0.22 ± 0.03
Mn_3.75_Ni_0.25_N	Mn Ni	0.081 ± 0.020 0.17 ± 0.02	0.011 ± 0.005 0.010 ± 0.002	0.092 ± 0.025 0.18 ± 0.02

**Figure 5.**
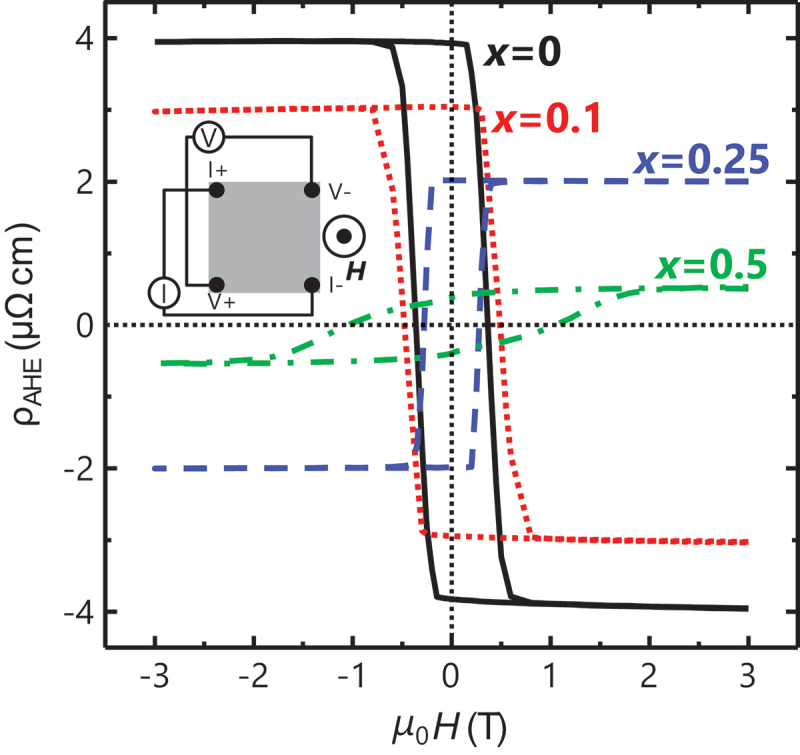
AHE loops measured at RT for Mn_4−*x*_Ni_*x*_N (*x* = 0, 0.1, 0.25, 0.5) films on STO (001). A magnetic field was applied normal to the sample plane [62]. Reproduced by permission from [62], copyright [2019, AIP Publishing].

Figure 5 shows the dependence of $\rho_{\mathrm{AHE}}$ on the perpendicular magnetic field for Mn_4−*x*_Ni_*x*_N epitaxial films (*x* = 0, 0.10, 0.25, and 0.5) on STO (001) at RT [62]. For the *x* = 0, 0.10, and 0.25 samples, the squareness ratio was almost 1. The sign of $\rho_{\mathrm{AHE}}$ reversed between *x* = 0.10 and 0.25. This shows that the relative orientation of the spin polarization and the magnetic moment changed between *x* = 0.10 and 0.25, confirming the crossover in the MC composition. Researchers generally use such a sign reversal to confirm the presence of MC in rare-earth ferrimagnets [12,13]. When the *x* was increased further to *x* = 0.5, a much greater magnetic field was required to saturate the magnetization, probably due to a degradation in crystal quality.

**Figure 6.**
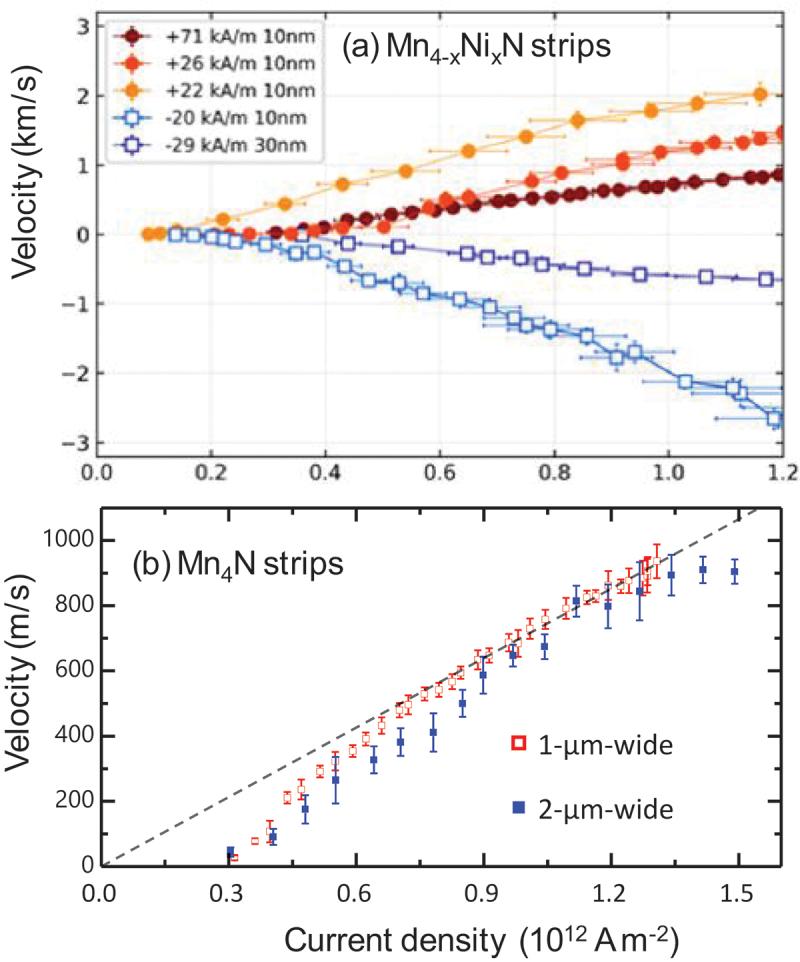
(a) Domain wall (DW) speed against current density for Mn_4−*x*_Ni_*x*_N strips for various Ni concentrations on either side of the MC composition. *M*_S_ = 71 kA m^−1^ corresponds to Mn_4_N (*x* = 0). The filled/empty symbols show the DW velocity below/above the MC composition: the direction of DW motion depends on whether the Ni composition is below or above the MC composition [19]. Reproduced by permission from [19], copyright [2021, American Chemical Society]. (b) DW velocity versus current density measured for Mn_4_N strips. The DW velocity reaches an average above 900 m s^−1^ at 1.3 × 10^12^ A/m^2^. The dotted line is a guide to the eye [45]. Reproduced by permission from [45], copyright [2019, American Chemical Society].

As mentioned above, Mn_4_N epitaxial films containing a small amount of Ni exhibit a decrease in magnetization, which approaches zero at RT near *x* = 0.1. In line with the reduction in magnetization of Mn_4−*x*_Ni_*x*_N, extremely fast current-induced domain wall (DW) motion driven purely by STT, exceeding 3,000 m s^−1^, was demonstrated in Mn_4−*x*_Ni_*x*_N microstrips at RT near the *x*_MC_ [19]. Figure 6(a) shows the relationship between DW velocity and current density in Mn_4−*x*_Ni_x_N microstrips (*x* = 0, 0.1, 0.15, 0.2, 0.25) with different Ni concentrations on either side of the *x*_MC_. For comparison, the results for Mn_4_N microstrips are also shown in Figure 6(b) [45]. In Figure 6(a), for MC compositions of *x* ≤ 0, 0.1, 0.15, and 0.2, the DW moved in the direction of the electron flow, and its mobility increased with increasing Ni composition. However, when the value of *x* exceeded 0.25, the direction of DW motion reversed, and at a current density of *j* = 1.2 × 10^12^ A m^−2^, a large *v*_DW_ of up to 3000 m s^−1^ relative to the current flow direction was obtained. This high mobility, obtained in Mn_4−*x*_Ni_*x*_N microstrips near the *x*_MC_, exceeded the highest velocity obtained by SOT driving in the absence of an external magnetic field in a ferromagnetic Pt/GdCo thin film at 250 K [14]. In these materials, the *x*_MC_ and the angular momentum compensation composition (*x*_AC_) are different. In contrast, in Mn_4_N, the two magnetic sublattices consist of Mn, Mn(I), and Mn(II). Considering that the Ni composition in Mn_4−*x*_Ni_*x*_N thin films is only about 5 at% of Mn(I) atoms, it can be assumed that the gyromagnetic factors of Mn(I) and Mn(II) are the same. As a result, the *x*_MC_ and the *x*_AC_ coincide.

### 3.3. Non-magnetic compensation in Cr-doped Mn_4_N epitaxial films

**Figure 7.**
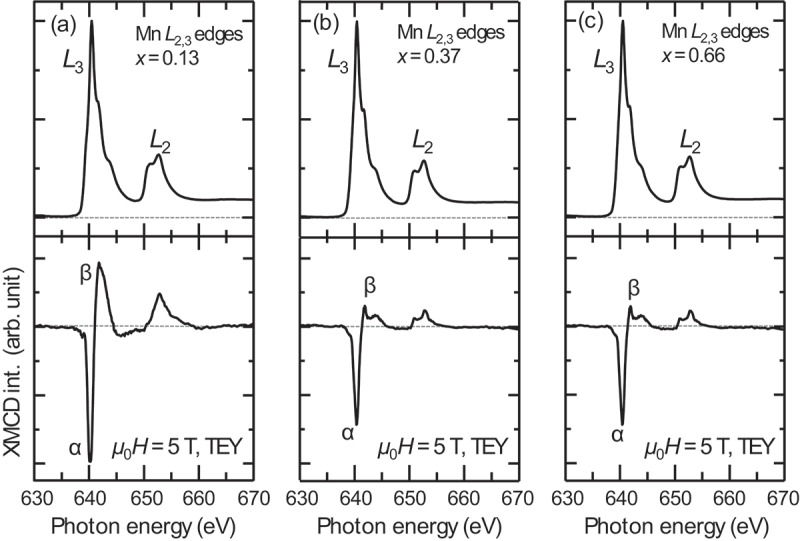
XAS and XMCD spectra measured measured at the Mn-*L*_2,3_ edges in Mn_4−*z*_Cr_*z*_N epitaxial films for (a) *z* = 0.13, (b) *z* = 0.37, and (c) *z* = 0.66 [63]. Reproduced by permission from [63], copyright [2022, Elsevier].

**Figure 8.**
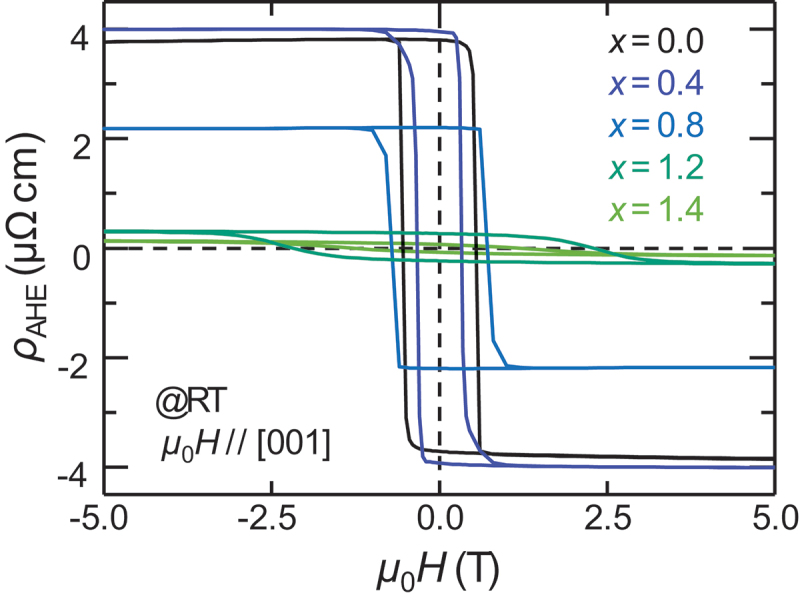
AHE loops of Mn4_−*z*_Cr_*z*_N epitaxial films [63]. Reproduced by permission from [63], copyright [2022, Elsevier].

Here, we present an example in which MC did not occur despite the introduction of transition metal impurities. This occurred in both Cr-doped Mn_4_N epitaxial films and Fe-doped Mn_4_N ones [63]. Figure 7 shows the XAS and XMCD spectra of Mn4_−*x*_Cr_*x*_N films (*x* = 0.13, 0.37, and 0.66) at the Mn-*L*_2,3_ edges [63]. Both an external magnetic field of up to *μ*_0_*H* = ± 5 T and circularly polarized soft X-rays with right or left polarization were applied at the so-called “magic angle of 54.7◦ to the sample normal [64–66]. At *x* = 0.13, the XAS and XMCD spectra at the Mn-*L*_2,3_ edges in Figure 7(a) were not significantly different from those of Mn_4_N in Figure 2. With increasing the Cr composition *x* further, the intensities of both peaks α and β decrease in Figure 7(b,c). This suggests that both the Mn(I) and Mn(II) sites were replaced by Cr atoms. No sign reversal of the XMCD spectrum, such as that observed in Mn_4−*x*_Ni_*x*_N, was observed. Figure 8 shows the AHE loops measured for Mn_4−*x*_Cr_*x*_N epitaxial films with various *x* values. Polarity reversal was not observed in the $\rho_{\mathrm{AHE}}$ loops within the measured Cr composition range. The difference in Ni and Cr atoms is the magnetic moment of these elements and their preference of site occupation. From an experimental perspective, the magnetic moment of the Mn(I) sublattice is parallel to the magnetization; consequently, its magnitude is greater than that of the Mn(II) sublattice. Therefore, it can be stated that an MC occurs when at least one of the following conditions is met.

- Substituting Mn(I) sites to reduce the magnetic moment of the Mn(I) sublattice.
- Substituting Mn(II) with magnetic moments increases the magnetic moment of the Mn(II) sublattice.

In the case of Mn_4−*x*_Ni_*x*_N films, MC occurred because the Mn(I) site was substituted with Ni, which has a magnetic moment antiparallel to that of Mn(I) atoms. In the case of Mn_4−*x*_Cr_*x*_N films, however, no preferred substitution site has been identified. Therefore, it falls into neither of the two categories described above.

### 3.4. Magnetic compensation in Mn_4_N epitaxial films using non-magnetic impurities (Cu, Ag, Au)

**Figure 9.**
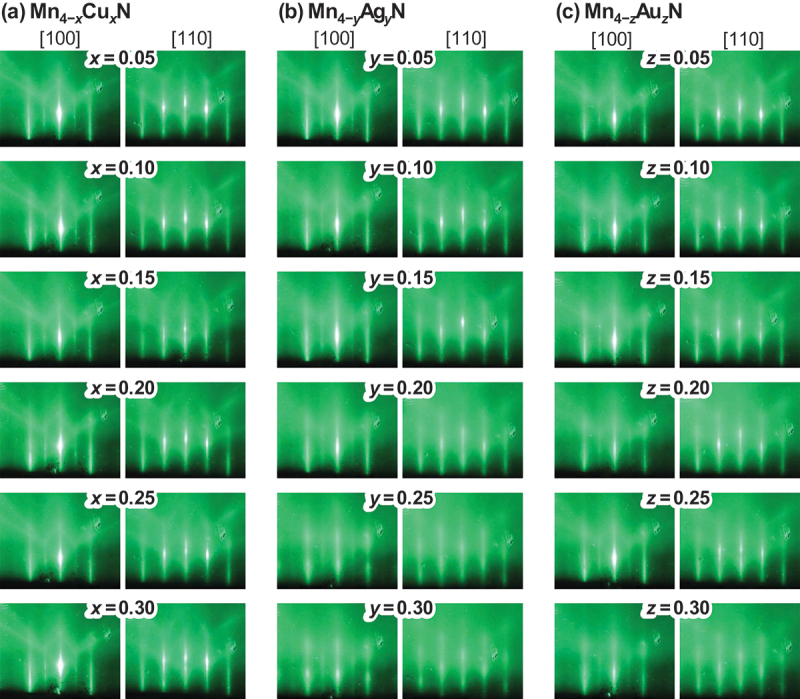
RHEED patterns measured on (a) Mn_4−*x*_Cu_*x*_N films, (b) Mn_4−_*_y_*Ag*_y_*N films, and (c) Mn_4−*z*_Au_*z*_N films, taken along the [100] and [1 1 0] azimuths of STO.

We next move on to Mn_4_N epitaxial films doped with non-magnetic elements and compare the results of doping three kinds of impurity atoms (Cu, Ag, and Au) in almost equal amounts. Figure 9(a–c) shows reflection high-energy electron diffraction (RHEED) patterns for 10-nm-thick Mn_4−*x*_Cu_*x*_N, Mn_4−_*_y_*Ag*_y_*N, and Mn_4−*z*_Au_*z*_N films, respectively, taken along the [100] and [1 1 0] azimuths of STO (001). The composition of impurity element was varied in the range 0.05–0.30. When the impurity composition was small, the streaky RHEED patterns with Kikuchi lines were observed regardless of impurity element, meaning that smooth epitaxial films were grown for all the Mn_4_N-based films. However, when the impurity composition was further increased to 0.30, differences began to appear on the type of impurity. Kikuchi lines disappeared except in Mn4_−*x*_Cu_*x*_N films, and the RHEED streaks lost their sharpness and became broad in Mn_4−_*_y_*Ag*_y_*N, and Mn_4−*z*_Au_*z*_N films.

**Figure 10.**
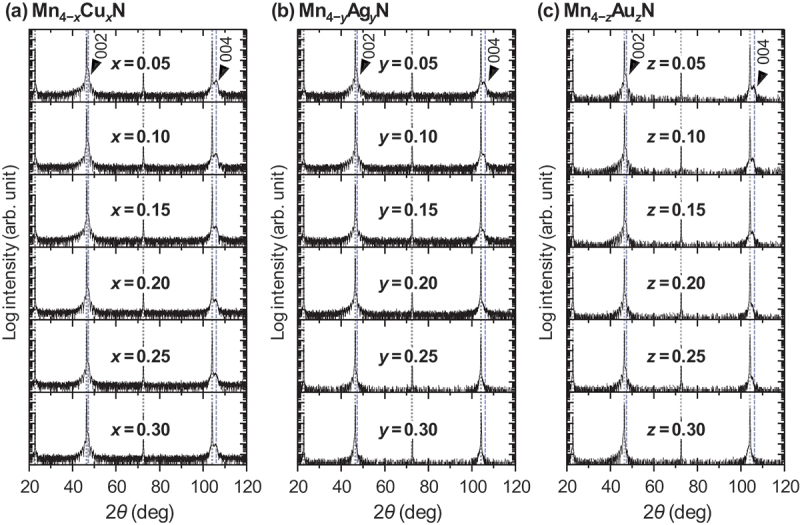
Out-of-plane XRD patterns for (a) Mn_4−*x*_Cu_*x*_N films, (b) Mn_4−_*_y_*Ag*_y_*N films, and (c) Mn_4−*z*_Au_*z*_N films.

**Figure 11.**
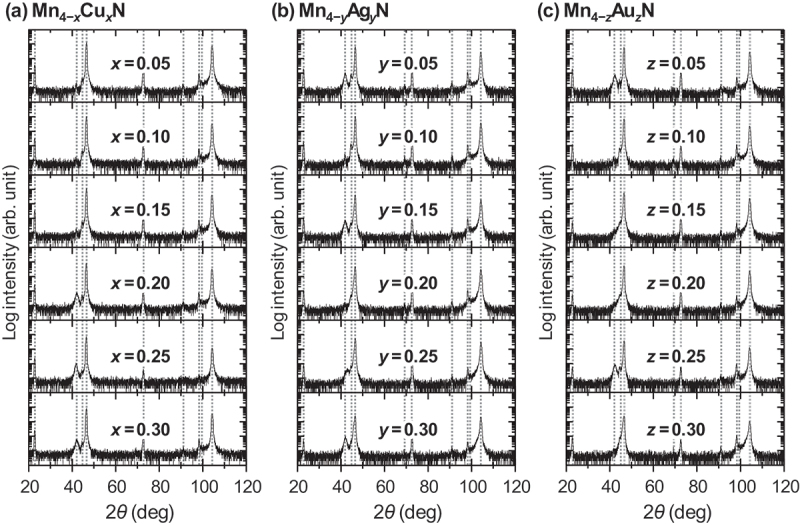
In-plane XRD patterns for (a) Mn_4−*x*_Cu_*x*_N films, (b) Mn_4−_*_y_*Ag*_y_*N films, and (c) Mn_4−*z*_Au_*z*_N films.

Figure 10(a–c) show the out-of-plane XRD patterns of Mn_4−*x*_Cu_*x*_N, Mn_4−_*_y_*Ag*_y_*N, and Mn_4−*z*_Au_*z*_N films, respectively. The 002 and 004 reflections of these films were observed as shown by close triangles. For Ag- and Au-doped Mn_4_N films, the peak position shifted toward lower 2*θ* angles as the doping concentration increases; at *y*, *z* = 0.30, it nearly overlapped with the substrate peak. These results indicate that <100>-oriented epitaxial films were grown for these samples. Figure 11(a–c) shows the in-plane XRD patterns of Mn_4−*x*_Cu_*x*_N, Mn_4−_*_y_*Ag*_y_*N, and Mn_4−*z*_Au_*z*_N films, respectively, with scattering vectors set along the [100] direction of the STO. In this case, almost no reflection from the epitaxial thin film was visible. This is likely because it overlapped with the peaks from the substrate.

**Figure 12.**
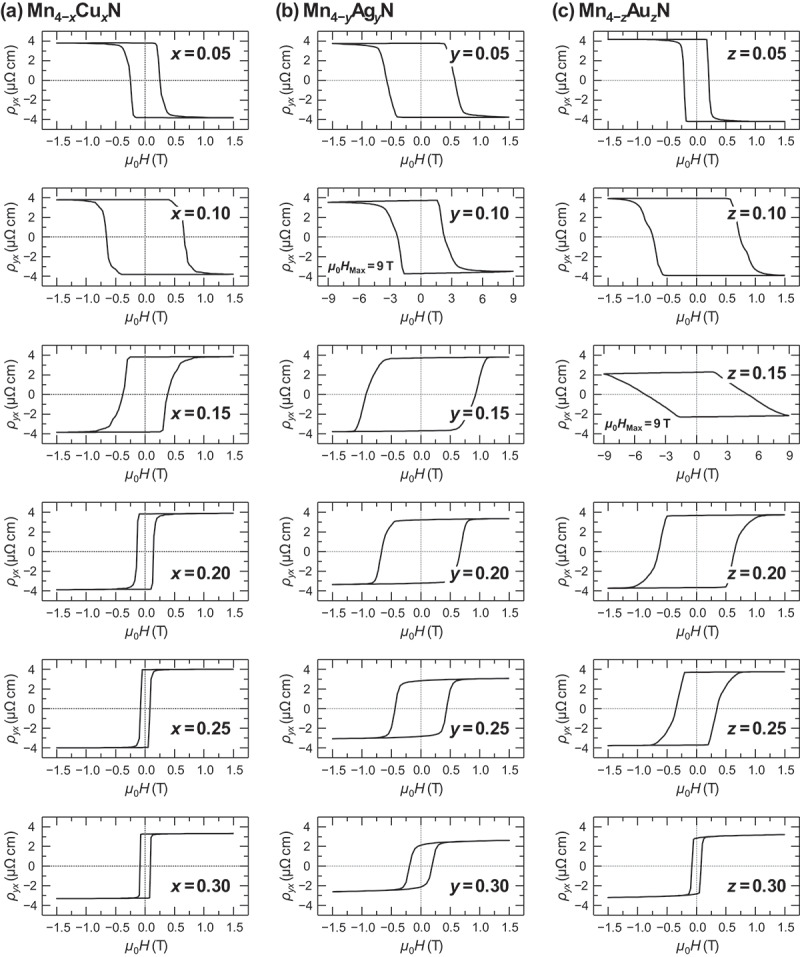
AHE loops of (a) Mn_4−*x*_Cu_*x*_N films, (b) Mn_4−_*_y_*Ag*_y_*N films, and (c) Mn_4−*z*_Au_*z*_N films.

Figure 12(a–c) show the AHE loops of Mn_4−*x*_Cu_*x*_N, Mn_4−_*_y_*Ag*_y_*N, and Mn_4−*z*_Au_*z*_N epitaxial films, respectively. For all samples, the coercive field increased with increasing impurity concentration and then decreased. Furthermore, at impurity concentrations where the coercive force was high, the sign of $\rho_{\mathrm{yx}}$ reversed. Based on the sign reversal in the AHE loops in the Mn_4−*x*_Ni_*x*_N thin films in Fig. 5 and the corresponding sign reversal in the XMCD spectra in Figure 4, we conclude that the MC occurs between *x*, *y* = 0.10 and 0.15 in the Mn_4−*x*_Cu_*x*_N [29] and Mn_4−_*_y_*Ag*_y_*N epitaxial films [30], and between *z* = 0.15 and 0.20 in the Mn_4−*z*_Au_*z*_N epitaxial films.

**Figure 13.**
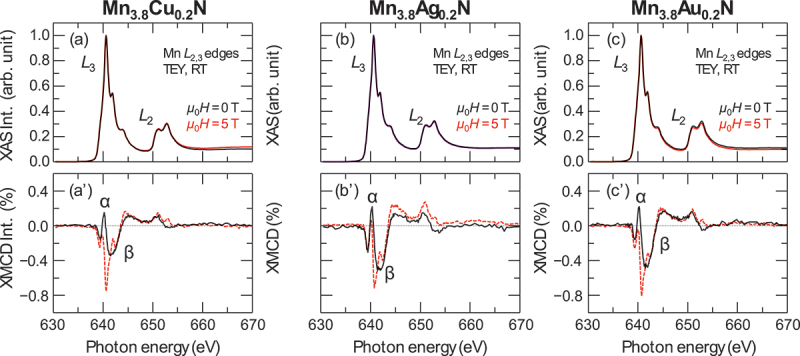
XAS and XMCD spectra at the Mn-*L*_2,3_ edges measured at 0 T and 5 T for (a)(a’) Mn_3.8_Cu_0.2_N, (b)(b’) Mn_3.8_Ag_0.2_N, and (c)(c’) Mn_3.8_Au_0.2_N. The spectrum at 0 T is obtained by first applying an external magnetic field up to 5 T, then lowering the field to 0 T, and finally performing the measurement.

**Figure 14.**
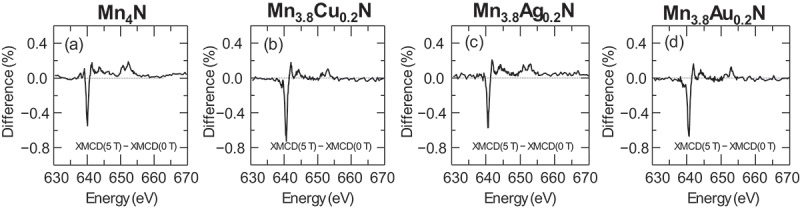
Differences in the XMCD spectra at the Mn-*L*_2,3_ edges for (a) Mn_4_N, (b) Mn_3.8_Cu_0.2_N, (c) Mn_3.8_Ag_0.2_N, and (d) Mn_3.8_Au_0.2_N epitaxial films, measured at 0 T and 5 T. The spectrum at 0 T is obtained by first applying an external magnetic field up to 5 T, then lowering the field to 0 T, and finally performing the measurement.

Figure 13(a–c) show the XAS and XMCD spectra of Mn_4−*x*_Cu_*x*_N, Mn_4−_*_y_*Ag*_y_*N, and Mn_4−*z*_Au_*z*_N epitaxial films, respectively, at *x*, *y*, *z* = 0.2. In all the samples, an external magnetic field of 0 T or 5 T and circularly polarized soft X-rays with right or left polarization were applied normal to the sample plane. It is noted that the spectra at 0 T were obtained by applying a magnetic field up to *μ*_0_*H* = ±5 T and then reducing it to *μ*_0_*H* = ±0 T. Based on the results in Figure 12, these impurity compositions are believed to exceed the *x*_MC_. At *μ*_0_*H* = 0 T, the XMCD spectra of the Mn_4−*x*_Cu_*x*_N, Mn_4−_*_y_*Ag*_y_*N, and Mn_4−*z*_Au_*z*_N epitaxial films were reversed compared to that of Mn_4_N epitaxial films. Therefore, it is concluded that the MC occurs in these three types of impurity-doped Mn_4_N epitaxial films. On the other hand, the XMCD spectra at *μ*_0_*H* = 5 T shown by the red dotted lines differ from those at *μ*_0_*H* = 0 T. Figure 14 shows the differences in the XMCD spectra at the Mn-*L*_2,3_ edges for (a) Mn_4_N, (b) Mn_3.8_Cu_0.2_N, (c) Mn_3.8_Ag_0.2_N, and (d) Mn_3.8_Au_0.2_N epitaxial films, measured at *μ*_0_*H* = 0 T and 5 T. Similar difference was also observed in the XMCD spectra for the Mn_4_N epitaxial films shown in Figure 14(a). Since such an unusual phenomenon did not appear in the electrical characteristics (AHE loops) shown in Figure 13, we suspect that it may be due to the diffusion of Mn into the SiO_2_ capping layers formed by sputtering. Therefore, to verify and discuss the sign reversal in the XMCD spectrum, it is better to saturate the magnetization in a high magnetic field and then lower the field to 0 T to conduct XAS and XMCD measurements, rather than comparing spectra under high magnetic fields.

### 3.5. Discussion on substitution sites

**Figure 15.**
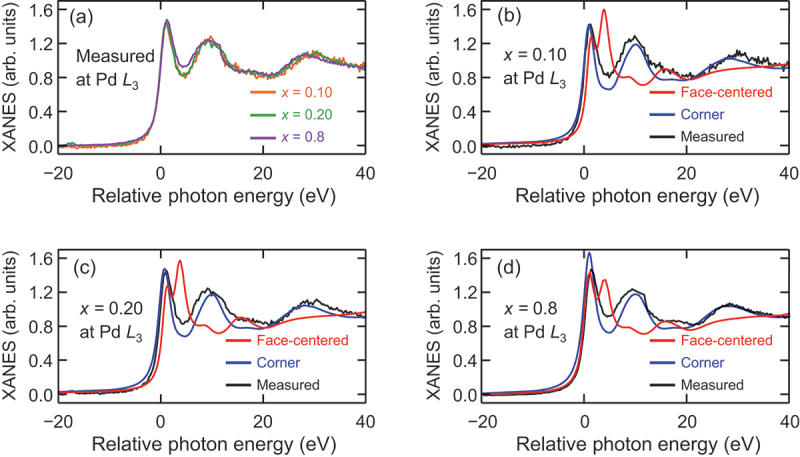
(a) XANES spectra of the Pd-*L*_3_ edge measured on Mn_4−*x*_Pd*_x_*N epitaxial films at *x* = 0.10 (orange), 0.20 (green), and 0.8 (purple). (b), (c), and (d) simulation and measurement results for the Pd *L*_3_-edge XANES spectra at *x* = 0.10, 0.20, and 0.8, respectively. The black lines are the measured spectra by the TFY method at RT [31]. The red and blue lines correspond to the simulation results for clusters, where the Pd atom are located at the face-centered and corner sites of the antiperovskite structures, respectively. Reproduced by permission from [31], copyright [2025, American Physical Society].

Regarding the substitution sites of impurities within the Mn_4_N film, XANES measurements were performed for Pd and Ag [30,31], and based on these results, it has been determined that the impurities occupy corner Mn(I) sites. Here, we present the results for Pd in Mn_4–_*ₓ*Pd*ₓ*N epitaxial films. Figure 15(a) shows the measured XANES spectra at the Pd *L*_3_ absorption edge for Mn_4–_*ₓ*Pd*ₓ*N (*x* = 0.10, 0.20, and 0.8). Even when the composition of Pd is changed, no change is observed in the peak positions of the spectrum [31]. This result suggests that the Pd substitutes for Mn at the same site in the three Pd compositions.

To compare these XANES spectra with the calculation results, XANES spectra were calculated for Mn_4−*x*_Pd_*x*_N using the FDMNES package. The calculations were performed for two structures: Mn_4_N-type cluster and Mn_3_PdN-type cluster. Both clusters included a center Pd as an absorbing atom. The difference between the two types is whether the central Pd atom is surrounded by a Mn_4_N crystal or a Mn_3_PdN crystal. Furthermore, we considered two substitutional sites for Pd: the corner and face-centered sites in the anti-perovskite structure in each case. To compare with experimental spectra, we adopted the spectra of the Mn_4_N-type cluster for a low-composition case (*x* = 0.1 and 0.2) and that of the Mn_3_PdN-type cluster for a high-composition case (*x* = 0.8). Prior to the comparison, we convoluted the simulated spectra using a Lorentzian function with an energy-dependent scale parameter, $\Gamma_{\mathrm{tot}}$. This can be expressed by the following equation.

(4) $$\begin{array}{rl} & \Gamma_{\mathrm{tot}}\left(E\right)=\Gamma_{\mathrm{hole}}+\Gamma_{\mathrm{electron}}\left(E\right)=\Gamma_{\mathrm{hole}}+\Gamma_{\max}\\ & \;\;\;\left(\frac{1}{2}+\frac{1}{\pi }\arctan\left(\frac{\pi }{3}\frac{\Gamma_{\max}}{E_{\mathrm{Larg}}}\left(e-\frac{1}{e^{2}}\right)\right)\right). \end{array}$$

(5) $$\begin{array}{c} e=\frac{E-E_{\mathrm{Fermi}}}{E_{\mathrm{cent}}}. \end{array}$$

Here, E is the photon energy, $\Gamma_{\mathrm{hole}}$ is the core-hole width, $\Gamma_{\mathrm{electron}}\left(E\right)$ is the photo-electron width, $\Gamma_{\max}$ is the maximum width of the final state, $E_{\mathrm{Larg}}$ is the width of the arctan function, $E_{\mathrm{cent}}$ is the center position of the arctan function, and $E_{\mathrm{Fermi}}$ is the Fermi energy. Three parameters, $E_{\mathrm{Fermi}}$, $\Gamma_{\mathrm{hole}}$, and $\Gamma_{\max}$, were optimized using an FDMNES code so that the experimental spectra matched the calculated spectra for the cluster with a Pd atom that substituted Mn at corner sites. In contrast, we also tried to fit the calculated spectra for face-centered Pd, the fitting code did not settle. Therefore, the same parameters were applied to the convolution. Table 2 summarizes the optimized parameters for the convolution. The optimization was conducted with the default parameters of $E_{\mathrm{cent}}=$ 30 eV, and $E_{\mathrm{Larg}}=$ 30 eV.

**Table 2.** Optimized parameters for the convolution of simulated spectra for FDMNES.

Pd composition *x*	$E_{\mathrm{Fermi}}$ (eV)	$\Gamma_{\mathrm{hole}}$ (eV)	$\Gamma_{\max}$ (eV)
0.1	0.17550	2.17875	15.42221
0.2	0.02606	2.33489	12.98211
0.8	0.05017	2.60336	12.99420

Figure 15(b–d) shows a comparison of the experimental results at *x* = 0.10, 0.20, and 0.8 with the XANES spectra predicted under the assumption that Pd has substituted for the corner Mn(I) sites or the face-centered Mn(II) sites. The convolution parameters of the calculated spectra were optimized with reference to the experimental data. The energy on the horizontal axis is normalized to 3,173 eV, which is the Pd *L*_3_ absorption edge. When Pd substitutes for a face-centered Mn(II) site, the simulated spectrum exhibits split peaks in the energy range from 0 to 7 eV. On the other hand, when Pd substitutes for a corner Mn(I) site, only a single peak is observed in the same energy range. Furthermore, in the energy region above 7 eV, the simulation corresponding to Pd substitution at the corner sites shows a high degree of agreement with the experimental data for the three simulated Pd compositions. The agreement between the experimental and simulated spectra was evaluated using the *D*_1_ factor, a measure commonly used to assess spectral similarity. A smaller *D*_1_ coefficient indicates a higher degree of agreement between the experimental and simulated spectra. The *D*_1_ factor is given by Eqs. (6) and (7):

(6) $$D_{1}=\frac{1}{2}\int^{\left|\frac{1}{c_{\mathrm{sim}}}I_{\mathrm{sim}}\left(E\right)-\frac{1}{c_{\exp}}I_{\exp}\left(E\right)\right|}\mathrm{dE}.$$

(7) $$c_{\mathrm{sim}}=\overset{E_{\max}}{\underset{E_{\min}}{\int }}I_{\mathrm{sim}}\left(E\right)\mathrm{dE},c_{\exp}=\overset{E_{\max}}{\underset{E_{\min}}{\int }}I_{\exp}\left(E\right)\mathrm{dE}.$$

**Figure 16.**
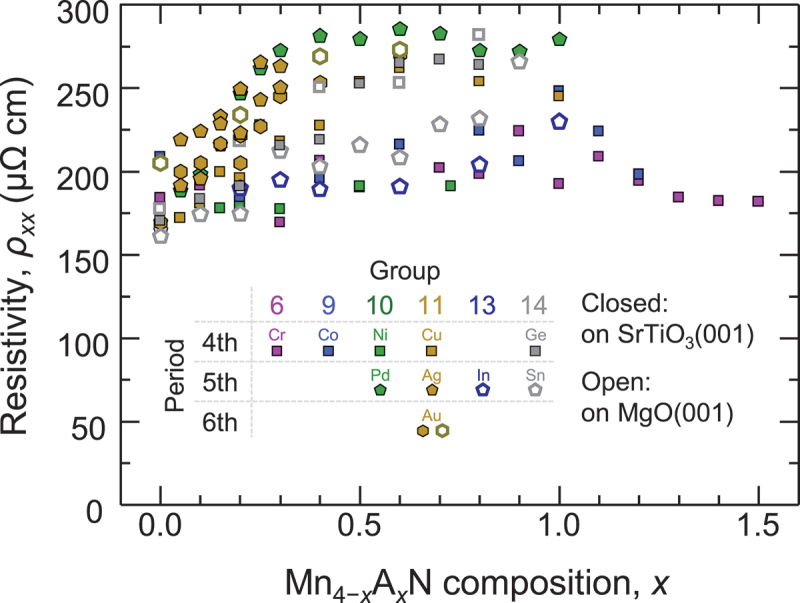
Longitudinal resistivity of impurity-doped Mn_4_N epitaxial films.

Here, $c_{\mathrm{sim}}$ and $c_{\exp}$ represent the integral of the simulation and experimental spectra, respectively, over the energy range between the minimum energy ($E_{\min})$ and maximum energy $\left(E_{\max}\right)$ under consideration. $I_{\mathrm{sim}}\left(E\right)$ and $I_{\exp}\left(E\right)$ represent the simulated and experimental spectra, respectively, at a given energy E. For the three Pd compositions examined, the *D*_1_ values obtained assuming that Pd substituted for corner Mn(I) sites were smaller than those obtained assuming that Pd substituted for face-centered Mn(II) sites as shown in Table 3. We therefore conclude that the Pd atoms preferentially substitute for Mn(I) sites in Mn_4−*x*_Pd_*x*_N epitaxial films in the range of *x* = 0.10–0.8. Similar results were obtained for Ga and Zn doped in Mn_4_N epitaxial films. In impurity-doped Mn_4_N epitaxial films, the impurities that cause MC at RT are currently Ni, Co, Cu, Ag, Au, and Pd, while Zn and Ga are under investigation, it can be stated that they preferentially substitute for the corner Mn(I) sites. A common feature of these impurity elements is that MC occurs at RT at doping levels of approximately 5 at%. Figure 16 shows the longitudinal electrical resistivity ($\rho_{\mathrm{xx}}$) of Mn_4_N epitaxial films doped with Cr, Co, Ni, Pd, Cu, Ag, Au, In, Ge, and Sn, measured at RT. Regardless of the type of impurity, as doped impurities are increased, the electrical resistivity gradually increases compared to that of Mn_4_N, and then reaches a plateau. Looking ahead to device applications, it is not possible to determine which impurity element is preferable based on Mn_4_N layers alone. When using SOT, for example, it is necessary to form a steep heterostructure with heavy metals (Pt, W). In such a structure, it is essential to identify a fabrication process that suppresses the diffusion of constituent elements, including impurity elements.

**Table 3.** *D*_1_ factors calculated from experimental and simulated spectra.

Nominal Pd composition (*x*)	*D*_1_ for corner simulation spectra	*D*_1_ for face-centered simulation spectra
0.10	3.281151	5.622976
0.20	2.726135	5.314904
0.8	2.832384	4.792633

### 3.6. Toward Mn_4_N on <100>-oriented Pt

Starting with a Ni-doped Mn_4_N film [26], we have grown Mn_4_N epitaxial thin films doped with various impurity elements and investigated whether they exhibit MC at RT. When applying Mn_4_N to devices as a next step, we aim to utilize the SOT that arises from a heterojunction with a heavy metal, and realize the layered structure of Mn_4_N/Pt/MgO (001). The reason for using Pt as an underlayer rather than a capping layer is to enable the formation of device structures, such as magnetic tunnel junctions (MTJ), on top of the Mn_4_N film. We also selected MgO rather than STO since MgO is commonly used for MTJs. The lattice constant of face-centered cubic Pt is 0.392 nm, which is close to that of Mn_4_N (0.387 nm). Therefore, we believe that if a Pt film with a <100> orientation can be formed on MgO, a Mn_4_N film can be epitaxially grown on top of it. When Pt is selected as the heavy metal [67–70], the key step is to form a <100>-oriented ultrathin Pt epitaxial film on MgO with a (001) surface. <100>-oriented Pt films are preferred compared to <111>-oriented films because they exhibit higher SOT efficiency via SHE [71]. The thickness of heavy-metal thin films used as spin sources is typically less than 10 nm. This is because the spin current generated by the SHE originates primarily from the region within the spin diffusion length (typically 10 nm or less) near the interface [72–76]. When the film is thicker than this length scale, the charge current flowing far from the interface contributes little to the generation of spin current; therefore, as the film thickness increases, the spin torque efficiency per unit current decreases [77,78]. However, reports of ⟨100⟩-oriented epitaxial Pt films that satisfy both continuity and smoothness have been limited to films thicker than 10 nm. This is because Pt films with (111) surfaces are the most stable [79–81].

**Figure 17.**
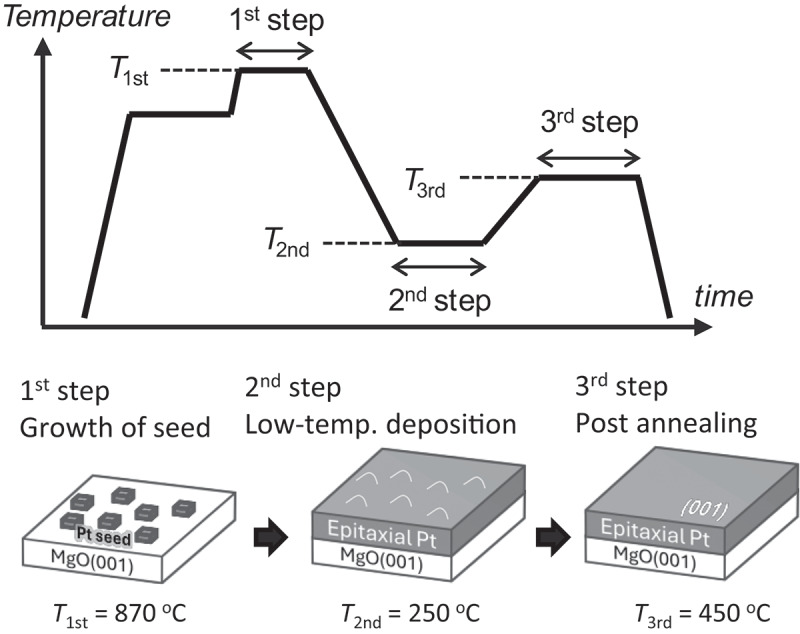
Schematic diagram of the 3-step growth process for high-quality <100>-oriented Pt films. The first step is the formation of seed crystals at *T*_1st_ = 870°C to establish <100> orientation, the second step is the Pt deposition at *T*_2nd_ = 250°C to ensure film continuity, and the third step is post-annealing at *T*_3rd_ = 450°C to improve surface flatness.

**Figure 18.**
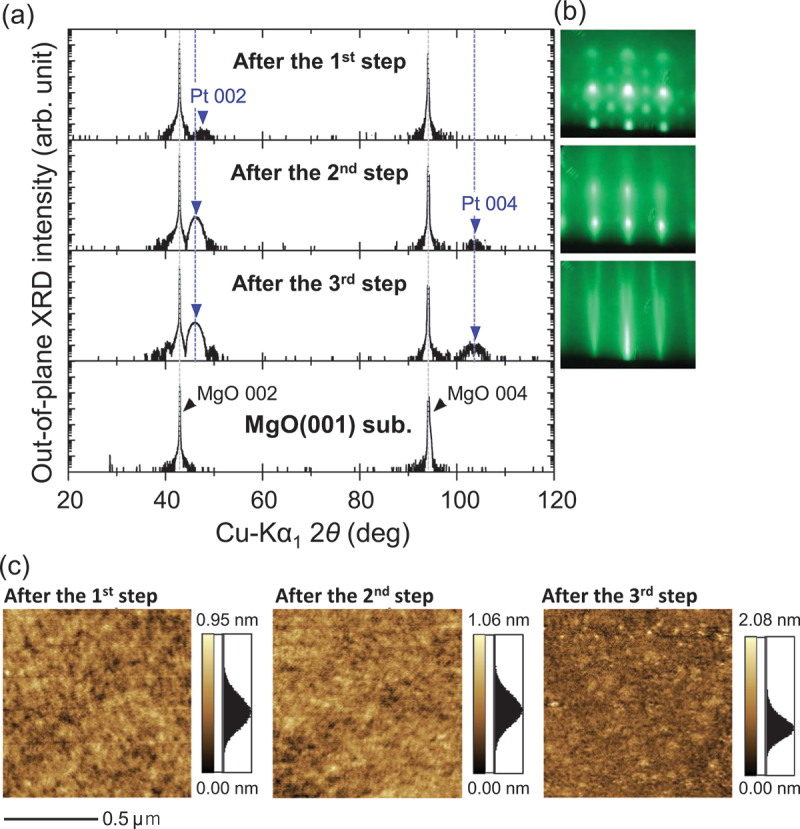
(a) Out-of-plane XRD patterns for Pt formed by the three-step growth process. The patterns are measured after the first step (growth of seed), the second step (low-temperature deposition), and the third step (post annealing). (b) RHEED patterns taken along the [100] azimuth. (c) AFM images of samples after each step.

We have addressed these challenges using a three-step growth method and have recently demonstrated the epitaxial growth of <100>-oriented Pt layers with a thickness of approximately 4 nm on MgO (001) substrates by radio-frequency sputtering [82]. Figure 17 shows a diagram of the three-step growth process of the Pt layers. The deposition rate of Pt was 0.23 nm min^−1^. Upon post-annealing, samples were heated to the target temperature at a rate of approximately 10°C min^−1^, then held at that temperature for 30 min under high vacuum. Samples were then cooled down to RT in the vacuum chamber. In the first step, Pt crystals oriented in the <100> direction were formed for 1.5 min at a substrate temperature of *T*_1st_ = 870°C, followed by the Pt deposition for 13.5 min at a substrate temperature of *T*_2nd_ = 250°C in the second step. In the third step, post annealing was performed at a substrate temperature of *T*_3rd_ = 450°C. The Pt deposited in the second step inherited the crystal orientation and roughness from the seed crystals formed in the first step as shown in Figure 18(a,b). Normally, when Pt is deposited on an MgO (001) substrate at 250°C, <111>-oriented Pt is formed [82]; however, it is important to note that because <100>-oriented Pt seed crystals are formed in the first step, even when Pt is deposited at 250°C in the second step, the resulting structure retains the <100> orientation. After the third step, RHEED streaks appeared, indicating the improved surface flatness. The 1/5-period RHEED streaks taken along the [100] direction originate from a Pt (001) − (5 × *n*) surface reconstruction [83–85]. AFM images in Figure 18(c) show that the surface roughness after the third step was significantly smaller and the formation of the <100>-oriented ultrathin continuous Pt films was confirmed. The longitudinal electrical resistance of the Pt layer was 24 µΩ cm at RT. The development of such ultra-thin Pt films described in this paper will facilitate the realization of highly energy-efficient Mn_4_N-based SOT devices on MgO with a (001) surface.

## 4. Conclusions

This paper summarizes experimental results regarding the potential of Mn_4_N thin films – ferrimagnetic materials containing no rare earth elements – with a particular focus on the presence or absence of MC at RT due to impurity doping. The presence of MCs at RT has been confirmed in Mn_4_N epitaxial films doped with Ni, Co, Cu, Ag, Au, and Pd. In Ni-doped Mn_4_N near the MC composition, current-induced domain wall motion driven solely by STT is realized at speeds exceeding 3000 m/s at RT, suggesting that the introduction of impurity elements that surpass Ni is expected. To further advance applications based on STT and SOT using Mn_4_N, it is necessary to study several physical properties in greater detail, such as spin current polarization and decay parameters. Furthermore, to utilize and enhance spin-orbit effects such as SOT and the Dzyaloshinskii Moriya interaction, it is essential to deposit ultrathin films of extremely high-quality Mn_4_N-based ferrimagnetic/heavy-metal structures. We have also demonstrated the formation of ultra-thin <100>-oriented Pt epitaxial film (approximately 4 nm in thickness) that serves as the foundation for such structures. This material development will help demonstrate the potential of Mn_4_N, extending beyond DW manipulation via pure STT to applications ranging from SOT to skyrmions and THz switching. We therefore hope that many researchers will take an interest in utilizing this material.

## Data Availability

The data will be available on request from the corresponding author.

## References

[1] Dieny B, Prejbeanu IL, Garello K, et al. Opportunities and challenges for spintronics in microelectronics industries. Nat Electron. 2020;3(8):446. doi: 10.1038/s41928-020-0461-5

[2] Shao QM, Li P, Liu LQ, et al. Roadmap of spin-orbit torques. IEEE Trans Magn. 2021;57(7):1. doi: 10.1109/TMAG.2021.3078583 PMC10091395 37057056

[3] Krizakova V, Perumkunnil M, Couet S, et al. Spin-orbit torque switching of magnetic tunnel junctions for memory applications. J Mag. 2022;562:169692. doi: 10.1016/j.jmmm.2022.169692

[4] Worledge DC, Hu GH. Spin-transfer torque magnetoresistive random access memory technology status and future directions. Nat Rev Elec Eng. 2024;1(11):730. doi: 10.1038/s44287-024-00111-z

[5] Stanciu CD, Kimel AV, Hansteen F, et al. Ultrafast spin dynamics across compensation points in ferrimagnetic GdFeCo: the role of angular momentum compensation. Phys Rev B. 2006;73:220402(R). doi: 10.1103/PhysRevB.73.220402

[6] Binder M, Weber A, Mosendz O, et al. Magnetization dynamics of the ferrimagnet CoGd near the compensation of magnetization and angular momentum. Phys Rev B. 2006;74(13):134404. doi: 10.1103/PhysRevB.74.134404

[7] Stanciu CD, Tsukamoto A, Kimel AV, et al. Rasing, all-optical magnetic recording with circularly polarized light. Phys Rev Lett. 2007;99:217207. doi: 10.1103/PhysRevLett.99.047601 17678404

[8] Fukami S, Suzuki T, Nakatani Y, et al. Current-induced domain wall motion in perpendicularly magnetized CoFeB nanowire. Appl Phys Lett. 2011;98:082504. doi: 10.1063/1.3558917

[9] Yang SH, Ryu KS, Parkin S. Domain-wall velocities of up to 750 m s^−1^ driven by exchange-coupling torque in synthetic antiferromagnets. Nat Nanotechnol. 2015;10:221. doi: 10.1038/nnano.2014.324 25705867

[10] Okuno T, Kim KJ, Tono T, et al. Temperature dependence of magnetoresistance in GdFeCo/Pt heterostructure. Appl Phys Express. 2016;9:073001. doi: 10.7567/APEX.9.073001

[11] Yu J, Qiu X, Wu Y, et al. Spin orbit torques and Dzyaloshinskii-Moriya interaction in dual-interfaced Co-Ni multilayer. Sci Rep. 2016;6:1. doi: 10.1038/srep32629 27601317 PMC5013523

[12] Kim KJ, Won SK, Hirata Y, et al. Fast domain wall motion in the vicinity of the angular momentum compensation temperature of ferrimagnets. Nat Mater. 2017;16:1187. doi: 10.1038/nmat4990 28967917

[13] Hirata Y, Kim DH, Okuno T, et al. Correlation between compensation temperatures of magnetization and angular momentum in GdFeCo ferrimagnets. Phys Rev Rev B. 2018;97:220403(R). doi: 10.1103/PhysRevB.97.220403

[14] Caretta L, Mann M, Buttner F, et al. Fast current-driven domain walls and small skyrmions in a compensated ferrimagnet. Nat Nanotechnol. 2018;13:1154. doi: 10.1038/s41565-018-0255-3 30224795

[15] Oh SH, Kim SK, Lee DK, et al. Coherent terahertz spin-wave emission associated with ferrimagnetic domain wall dynamics. Phys Rev B. 2017;96:100407(R). doi: 10.1103/PhysRevB.96.100407

[16] Siddiqui AS, Han J, Finley JT, et al. Current-induced domain wall motion in a compensated ferrimagnet. Phys Rev Lett. 2018;121:057701. doi: 10.1103/PhysRevLett.121.057701 30118301

[17] Blasing R, Ma T, Yang SH, et al. Exchange coupling torque in ferrimagnetic Co/Gd bilayer maximized near angular momentum compensation temperature. Nat Commun. 2018;9:4984. doi: 10.1038/s41467-018-07373-w 30478261 PMC6255835

[18] Cai K, Zhu Z, Lee JM, et al. Ultrafast and energy-efficient spin–orbit torque switching in compensated ferrimagnets. Nat Electron. 2020;3:37. doi: 10.1038/s41928-019-0345-8

[19] Ghosh S, Komori T, Hallaj A, et al. Current-driven domain wall dynamics in ferrimagnetic nickel-doped Mn_4_N films: very large domain wall velocities and reversal of motion direction across the magnetic compensation point. Nano Lett. 2021;21:2580. doi: 10.1021/acs.nanolett.1c00125 33705154

[20] Li Y, Zheng DX, Liu C, et al. Current-induced magnetization switching across a nearly room-temperature compensation point in an insulating compensated ferrimagnet. ACS Nano. 2022;16:8181. doi: 10.1021/acsnano.2c01788 35549072

[21] Zhu L, Ralph DC. Strong variation of spin-orbit torques with relative spin relaxation rates in ferrimagnets. Nat Commun. 2023;14:1778. doi: 10.1038/s41467-023-37506-9 36997579 PMC10063689

[22] Zhu W, Tang M, Pan C, et al. Sign-tunable magnetic tunnel junctions engineered via ferrimagnets for efficient all-electrical and thermal switching. Adv Func Mater. 2026;36(12). doi: 10.1002/adfm.202505415

[23] Duan Z, Smith A, Yang L, et al. Nanowire spin torque oscillator driven by spin orbit torques. Nat Commun. 2024;5:5616. doi: 10.1038/ncomms6616 25476073

[24] Finley J, Lee CH, Huang PY, et al. Spin–orbit torque switching in a nearly compensated Heusler ferrimagnet. Adv Mater. 2019;31:1805361. doi: 10.1002/adma.201805361 30412315

[25] Mishra R, Yu J, Qiu X, et al. Anomalous current-induced spin torques in ferrimagnets near compensation. Phys Rev Lett. 2017;118:167201. doi: 10.1103/PhysRevLett.118.167201 28474947

[26] Komori T, Hirose T, Gushi T, et al. Magnetic reversal in rare-earth free Mn_4−x_Ni_x_N epitaxial films below and above Ni composition needed for magnetic compensation around room temperature. J Appl Phys. 2020;127:043903. doi: 10.1063/1.5128635

[27] Mitarai H, Hirose T, Ito K, et al. Magnetic compensation at two different composition ratios in rare-earth-free Mn_4-*x*_Co*_x_*N ferrimagnetic films. Phys Rev Mater. 2020;4:094401. doi: 10.1103/PhysRevMaterials.4.094401

[28] Zhang R, He Y, Fruchart D, et al. Rare-earth-free noncollinear metallic ferrimagnets Mn_4-x_Z_x_N with compensation at room temperature. Acta Mater. 2022;234:118021. doi: 10.1016/j.actamat.2022.118021

[29] Hatate A, Yasuda T, Amemiya K, et al. Magnetic compensation in Mn_4−*x*_Cu*_x_*N films on SrTiO_3_(001) with noncollinear magnetic structures. Phys Rev Mater. 2024;8:L091403. doi: 10.1103/PhysRevMaterials.8.L091403

[30] Sobukawa Y, Yasuda T, Amemiya K, et al. Magnetic compensation and preferential substitution site for Ag in Mn_4−*x*_Ag*_x_*N epitaxial thin films. Phys Rev Mater. 2025;9:064408. doi: 10.1103/lx38-nzp9

[31] Akita S, Yasuda T, Amemiya K, et al. Mn_4−*x*_Pd*_x_*N epitaxial thin films: magnetic compensation at room temperature, Pd preferential site, and various magnetic properties. Phys Rev Mater. 2025;9:094411. doi: 10.1103/w6gs-wdq3

[32] Suemasu T, Vila L, Attané JP. Present status of rare-earth free ferrimagnet Mn_4_N and future prospects of Mn_4_N-based compensated ferrimagnets. J Phys Soc Jpn. 2021;90:081010. doi: 10.7566/JPSJ.90.081010

[33] Ito K, Honda S, Suemasu T. Transition metal nitrides and their mixed crystals for spintronics. Nanotech. 2022;33:062001. doi: 10.1088/1361-6528/ac2fe4 34649229

[34] Hirose T, Komori T, Gushi T, et al. Strong correlation between uniaxial magnetic anisotropy constant and in-plane tensile strain in Mn_4_N epitaxial films. AIP Adv. 2020;10:025117. doi: 10.1063/1.5141818

[35] Suemasu T. Rare-earth-free ferrimagnetic Mn_4_N spintronics. Bristol (UK): IOP Publishing; 2023. doi: 10.1088/978-0-7503-5477-6

[36] Yasutomi Y, Ito K, Sanai T, et al. Perpendicular magnetic anisotropy of Mn_4_N films on MgO(001) and SrTiO_3_(001) substrates. J Appl Phys. 2014;115:17A935. doi: 10.1063/1.4867955

[37] Kabara K, Tsunoda M. Perpendicular magnetic anisotropy of Mn_4_N films fabricated by reactive sputtering method. J Appl Phys. 2015;117:17B512. doi: 10.1063/1.4913730

[38] Ito K, Yasutomi Y, Kabara K, et al. Perpendicular magnetic anisotropy in Co*_x_*Mn_4-*x*_N (*x* = 0 and 0.2) epitaxial films and possibility of tetragonal Mn_4_N phase. AIP Adv. 2016;6:056201. doi: 10.1063/1.4942548

[39] Isogami S, Masuda K, Miura Y. Contributions of magnetic structure and nitrogen to perpendicular magnetocrystalline anisotropy in antiperovskite ε-Mn_4_N. Phys Rev Mater. 2020;4:014406. doi: 10.1103/PhysRevMaterials.4.014406

[40] He YK, Lenne S, Gercsi Z, et al. Noncollinear ferrimagnetism and anomalous Hall effects in Mn_4_N thin films. Phys Rev B. 2022;106:L060409. doi: 10.1103/PhysRevB.106.L060409

[41] Ching KM, Chang WD, Chin TS, et al. Anomalous perpendicular magnetoanisotropy in Mn_4_N films on Si(100). J Appl Phys. 1994;76:6582. doi: 10.1063/1.358200

[42] Ching KM, Chang WD, Chin TS. Magnetic properties and structure of Mn_4_N films on glass substrates. J Alloys Compd. 1995;222:184. doi: 10.1016/0925-8388(94)04914-9

[43] Shen X, Chikamatsu A, Shigematsu K, et al. Metallic transport and large anomalous Hall effect at room temperature in ferrimagnetic Mn_4_N epitaxial thin film. Appl Phys Lett. 2014;105:072410. doi: 10.1063/1.4893732

[44] Hirose T, Komori T, Gushi T, et al. Perpendicular magnetic anisotropy in ferrimagnetic Mn_4_N films grown on (LaAlO_3_)_0.3_(Sr_2_TaAlO_6_)_0.7_(001) substrates by molecular beam epitaxy. J Cryst Growth. 2020;535:125566. doi: 10.1016/j.jcrysgro.2020.125566

[45] Gushi T, Klug MJ, Garcia JP, et al. Large current driven domain wall mobility and gate tuning of coercivity in ferrimagnetic Mn_4_N thin films. Nano Lett. 2019;19:8716. doi: 10.1021/acs.nanolett.9b03416 31664840

[46] Burrowes C, Mihai AP, Ravelosona D, et al. Non-adiabatic spin-torques in narrow magnetic domain walls. Nat Phys. 2010;6:17. doi: 10.1038/nphys1436

[47] Emori S, Beach GSD. Enhanced current-induced domain wall motion by tuning perpendicular magnetic anisotropy. Appl Phys Lett. 2011;98:132508. doi: 10.1063/1.3570652

[48] Thiaville A, García JM, Miltat J. Domain wall dynamics in nanowires. J Magn Magn Mater. 2002;242–245:1061. doi: 10.1016/S0304-8853(01)01353-1

[49] Mougin A, Cormier M, Adam JP, et al. Domain wall mobility, stability and Walker breakdown in magnetic nanowires. Europhys Lett. 2007;78:57007. doi: 10.1209/0295-5075/78/57007

[50] Parveen F, He Z, Angizi S, et al. Hybrid polymorphic logic gate with 5-terminal magnetic domain wall motion device. IEEE Comput Soc Annu Symp VLSI (ISVLSI). 2017:152. doi: 10.1109/ISVLSI.2017.35

[51] Fukami S, Ohno H. Magnetization switching schemes for nanoscale three-terminal spintronics devices. Jpn J Appl Phys. 2017;56:0802A1. doi: 10.7567/JJAP.56.0802A1

[52] Parkin S, Hayashi M, Thomas L. Magnetic domain-wall racetrack memory. Science. 2008;320:190. doi: 10.1126/science.1145799 18403702

[53] Kawasaki M, Takahashi K, Maeda T, et al. Atomic control of the SrTiO_3_ crystal surface. Science. 1994;266:1540. doi: 10.1126/science.266.5190.1540 17841713

[54] Ohno H, Shen A, Matsukura F, et al. (Ga,Mn)As: a new diluted magnetic semiconductor based on GaAs. Appl Phys Lett. 1996;69:363. doi: 10.1063/1.118061

[55] Ravel B, Newville M. Athena, Artemis, Hephaestus: data analysis for X-ray absorption spectroscopy using IFEFFIT. J Synchrotron Radiat. 2005;12:537. doi: 10.1107/S0909049505012719 15968136

[56] Joly Y, Bunu O, Lorenzo JE, et al. Self-consistency, spin-orbit and other advances in the FDMNES code to simulate XANES and RXD experiments. J Phys Conf Ser. 2009;190:012007. doi: 10.1088/1742-6596/190/1/012007

[57] Ito K, Yasutomi Y, Zhu S, et al. Manipulation of saturation magnetization and perpendicular magnetic anisotropy in epitaxial Co*_x_*Mn_4−*x*_N films with ferrimagnetic compensation. Phys Rev B. 2020;101:104401. doi: 10.1103/PhysRevB.101.104401

[58] Thole BT, Carra P, Sette F, et al. X-ray circular dichroism as a probe of orbital magnetization. Phys Rev Lett. 1992;68:1943. doi: 10.1103/PhysRevLett.68.1943 10045260

[59] Carra P, Thole BT, Altarelli M, et al. X-ray circular dichroism and local magnetic fields. Phys Rev Lett. 1993;70:694. doi: 10.1103/PhysRevLett.70.694 10054179

[60] Nagai K, Fujiwara H, Aratani H, et al. Electronic structure and magnetic properties of the half-metallic ferrimagnet Mn_2_VAl probed by soft x-ray spectroscopies. Phys Rev B. 2018;97:035143. doi: 10.1103/PhysRevB.97.035143

[61] Kuch W, Gilles J, Kang SS, et al. Magnetic-circular-dichroism microspectroscopy at the spin reorientation transition in Ni(001) films. Phys Rev B. 2000;62:3824. doi: 10.1103/PhysRevB.62.3824

[62] Komori T, Gushi T, Anzai A, et al. Magnetic and magneto-transport properties of Mn_4_N thin films by Ni substitution and their possibility of magnetic compensation. J Appl Phys. 2019;125:213902. doi: 10.1063/1.5089869

[63] Komori T, Horiuchi T, Mitarai H, et al. Magnetic structure of 3*d*-element doped Mn_4_N films confirmed by X-ray magnetic circular dichroism – conditions for magnetic compensation. J Mag Mag Mater. 2022;564:170050. doi: 10.1016/j.jmmm.2022.170050

[64] Weller D, Stöhr J, Nakajima R, et al. Microscopic origin of magnetic anisotropy in Au/Co/Au probed with x-ray magnetic circular dichroism. Phys Rev Lett. 1995;75:3752. doi: 10.1103/PhysRevLett.75.3752 10059718

[65] Stöhr J, König H. Determination of spin- and orbital moment anisotropies in transition metals by angle-dependent x-ray magnetic circular dichroism. Phys Rev Lett. 1995;75:3748. doi: 10.1103/PhysRevLett.75.3748 10059717

[66] Koide T, Miyauchi H, Okamoto J, et al. Direct determination of interfacial magnetic moments with a magnetic phase transition in Co nanoclusters on Au(111). Phys Rev Lett. 2001;87:257201. doi: 10.1103/PhysRevLett.87.257201 11736602

[67] Hoffmann A. Spin hall effects in metals. IEEE Trans Magn. 2013;49(10):5172. doi: 10.1109/TMAG.2013.2262947

[68] Sinova J, Valenzuela SO, Wunderlich J, et al. Spin hall effects. Rev Mod Phys. 2015;87:1213. doi: 10.1103/RevModPhys.87.1213

[69] Zhu L, Ralph DC, Buhrman RA. Maximizing spin-orbit torque generated by the spin Hall effect of Pt. Appl Phys Rev. 2021;8:031308. doi: 10.1063/5.0059171

[70] Salemi L, Oppeneer PM. First-principles theory of intrinsic spin and orbital Hall and Nernst effects in metallic monoatomic crystals. Phys Rev Mater. 2022;6:095001. doi: 10.1103/PhysRevMaterials.6.095001

[71] Zhang Q, Zhao Y, He C, et al. Wang S, perpendicular magnetization switching driven by spin-orbit torque for artificial synapses in epitaxial Pt-based multilayers. Adv Electron Mater. 2022;8:2200845. doi: 10.1002/aelm.202200845

[72] Castel V, Vlietstra N, Ben Youssef J, et al. Thickness dependence of the inverse spin-Hall voltage from spin pumping in a hybrid yttrium iron garnet/platinum system. Appl Phys Lett. 2012;101:132414. doi: 10.1063/1.4754837

[73] Rojas-Sánchez JC, Reyren N, Garcia-Barriocanal J, et al. Spin pumping and inverse spin Hall effect in platinum: the essential role of spin-memory loss at metallic interfaces. Phys Rev Lett. 2014;112:106602. doi: 10.1103/PhysRevLett.112.106602 24679318

[74] Tao X, Liu Q, Miao B, et al. Self-consistent determination of spin Hall angle and spin diffusion length in Pt and Pd: the role of the interface spin loss. Sci Adv. 2018;4:eaat1670. doi: 10.1126/sciadv.aat1670 29942861 PMC6014716

[75] Sagasta E, Omori Y, Isasa M, et al. Tuning the spin Hall effect of Pt from the moderately dirty to the superclean regime. Phys Rev B. 2016;94:060412(R. doi: 10.1103/PhysRevB.94.060412

[76] Choi G, Ryu J, Thompson R, et al. Thickness dependence of spin–orbit torques in Pt/Co structures on epitaxial substrates. APL Mater. 2022;10:011105. doi: 10.1063/5.0077074

[77] Nguyen MH, Ralph DC, Buhrman RA. Spin torque study of the spin Hall conductivity and spin diffusion length in platinum thin films with varying resistivity. Phys Rev Lett. 2016;116:126601. doi: 10.1103/PhysRevLett.116.126601 27058088

[78] Mihajlović G, Mosendz O, Wan L, et al. Pt thickness dependence of spin Hall effect switching of in-plane magnetized CoFeB free layers studied by differential planar Hall effect. Appl Phys Lett. 2016;109:192404. doi: 10.1063/1.4967318

[79] Goniakowski J, Jelea A, Mottet C, et al. Structures of metal nanoparticles adsorbed on MgO(001). J Chem Phys. 2009;130:174703. doi: 10.1063/1.3121307 19425794

[80] Kozlov SM, Aleksandrov HA, Goniakowski J, et al. Effect of MgO(100) support on structure and properties of Pd and Pt nanoparticles with 49–155 atoms. J Chem Phys. 2013;139:084701. doi: 10.1063/1.4817948 24007023

[81] Seo H, Posadas AB, Demkov AA. First-principles study of the growth thermodynamics of Pt on SrTiO_3_ (001). J Vac Sci Technol B. 2012;30:04E108. doi: 10.1116/1.4732461

[82] Akita S, Yasuda T, Suemasu T. Epitaxial growth of <100>-oriented ultrathin (≃4 nm) continuous Pt films on MgO(001) and SrTiO_3_(001) substrates. ACS Appl Mater Interface. 2026;18:23868. doi: 10.1021/acsami.6c03772 41996669

[83] Zei MS, Batina N, Kolb DM. On the stability of reconstructed Pt(100) in an electrochemical cell: an ex-situ LEED/RHEED and in-situ STM study. Surf Sci. 1994;306(1–2):L519. doi: 10.1016/0039-6028(94)91171-1

[84] Zei MS, Ertl G. On the structural transformation of the reconstructed Pt(100) in electrolyte solutions. Surf Sci. 1999;442:19. doi: 10.1016/S0039-6028(99)00806-7

[85] Havu P, Blum V, Havu V, et al. Large-scale surface reconstruction energetics of Pt(100) and Au(100) by all-electron density functional theory. Phys Rev B. 2010;82:161418. doi: 10.1103/PhysRevB.82.161418

